# Tianwen-1 and China's Mars exploration program

**DOI:** 10.1093/nsr/nwab001

**Published:** 2021-01-16

**Authors:** Weijie Zhao

## Abstract

About every 26 months, the distance between Earth and Mars reaches a minimum, and that is the best time window for Mars exploration from Earth. In July 2020, three spacecraft started their journey to Mars: the Hope orbiter of the United Arab Emirates, the Tianwen-1 mission of China and the Perseverance rover of the United States’ National Aeronautics and Space Administration (NASA). If all go well, these spacecraft will reach Martian orbit in February 2021 and start their scientific observations.

Tianwen-1 is China's first mission to Mars. It includes an orbiter, a lander and a rover. It carries 13 scientific payloads and will investigate the topography, soil composition, water-ice distribution, internal structure, atmospheric environment and physical fields (electromagnetic and gravitational) of Mars. In this interview, we talked with the mission's Chief Scientist Yongxin Pan (潘永信) and Scientific Payload Sub-System Director Chi Wang (王赤) about this scientific mission and China's future plans for Mars exploration.

## Why do we explore Mars?

***NSR:*** In your opinion, what are the most significant scientific topics with regard to Mars?

***Pan:*** Mars is the neighbor of the Earth and these two planets share many similarities. So we are very interested in what Mars is like, and we also want to know what the processes were that made the red planet, which may once have been very similar to the Earth, but is now so different from our planet. We are also very interested in its habitability. Was there life? Were there oceans, a dense atmosphere and a global magnetic field, which are crucial for the existence of life? These are all significant scientific questions to be addressed.

***Wang:*** Within the solar system, Mars is the planet that resembles the Earth most. That is why we pay so much attention to it. Learning more about Mars can help us to better understand the Earth and the origin and evolution of the solar system.

Furthermore, Mars is the first candidate to become humanity's ‘second home’. NASA’s Mars exploration missions have always focused on the search for water and life. If we can find life or create a habitable environment on Mars, that would be a major breakthrough that could change the future of humankind.

***NSR:*** There are many descriptions about Mars, such as that there were once oceans on it, and now there is still water, and some people say that there may be life. Scientifically speaking, which descriptions have been confirmed and which are still hypotheses?

***Pan:*** It is quite certain that there is water-ice existing on Mars’s polar regions today. The ice sheets comprise mainly CO_2_-ice, but also contain water-ice. According to the radar results, there may also be a subsurface water-ice layer in some middle to lower latitude areas.

**Figure:**
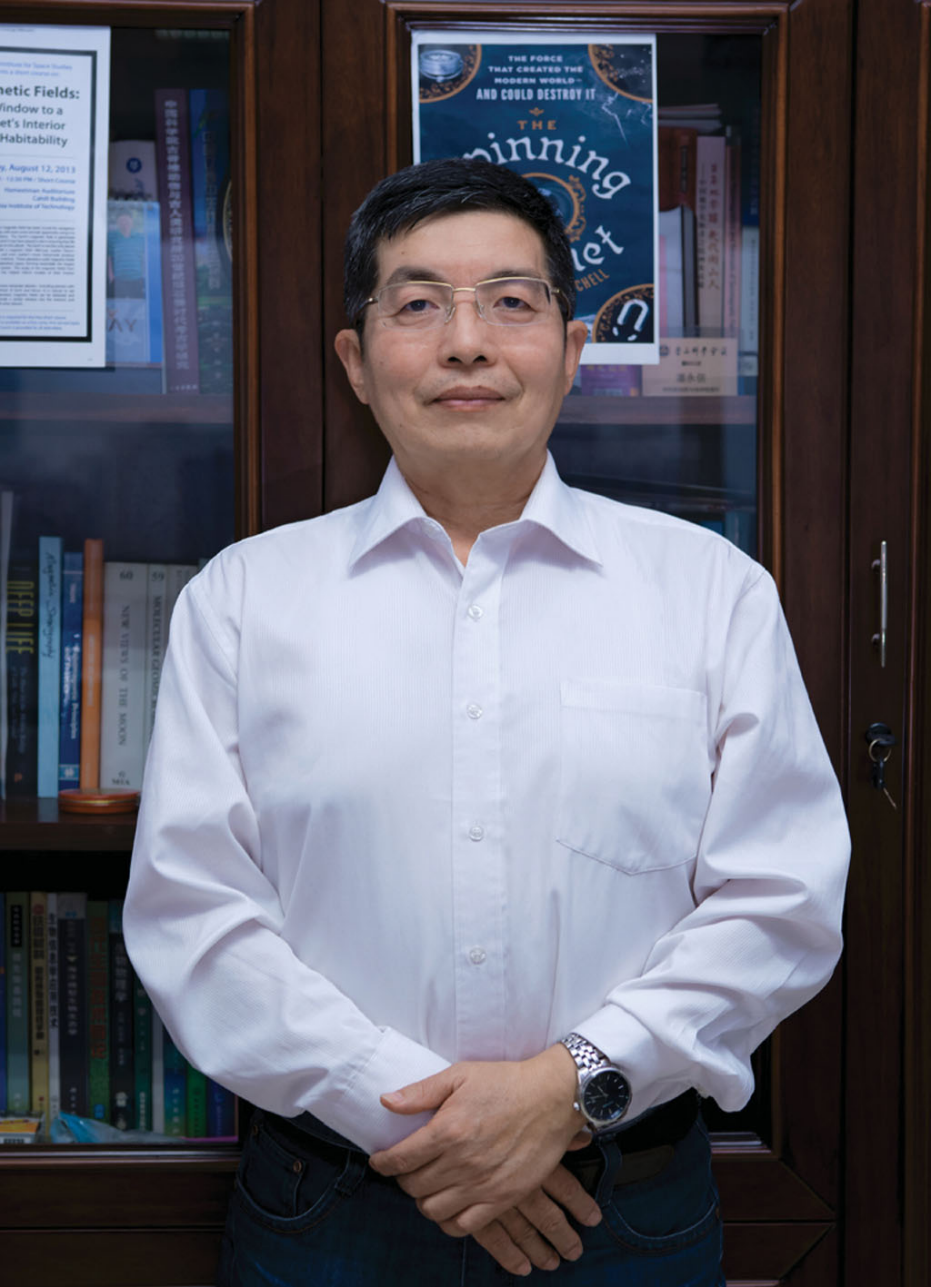
Yongxin Pan is the Chief Scientist of Tianwen-1 and a professor of the Institute of Geology and Geophysics, CAS *(Courtesy of Prof. Pan)*.

There is evidence that liquid water also exists on Mars today. For example, in 2018, an Italian research group reported that there is liquid water beneath the South Pole ice sheet. But the data and conclusions are still under debate.

Moreover, there has been a lot of evidence indicating that large amounts of liquid water once existed on Mars’s surface, which means that there were once ancient oceans and ancient rivers. Firstly, there are many geomorphic features of ancient river channels, ancient alluvial fans and ancient coastal zones existing on the northern hemisphere of Mars. Secondly, the D/H isotopic ratio of Martian water clearly indicates that there was once a large amount of water on Mars—but it has mostly escaped. Based on the isotope data, some researchers estimate that the ancient Martian ocean could have been as deep as 100 meters or more. Thirdly, there is a lot of evidence of sedimentary rocks and minerals, such as phyllosilicates, which are usually formed under aqueous environments. Supported by these lines of evidence, scientists have reached the consensus that there was once large amounts of liquid water on Mars. NASA’s Perseverance rover will land on a fossil river delta, where the possibility of finding signs of life is high.

**Figure:**
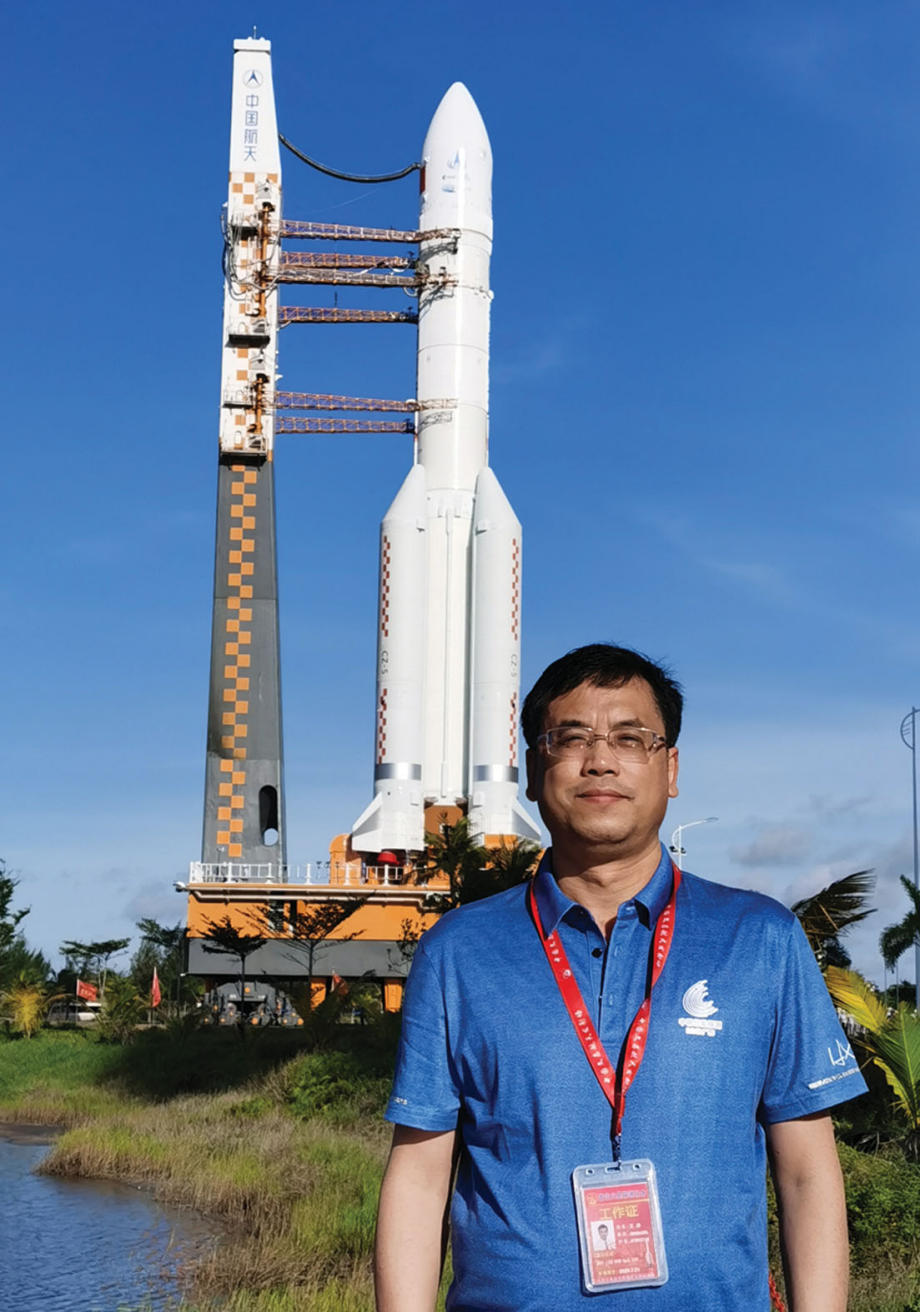
Chi Wang is the Scientific Payload Sub-System Director of Tianwen-1 and the Director General of the National Space Science Center, CAS. This photo was taken before the launch of Tianwen-1 with the Long March 5 rocket as background *(Courtesy of Prof. Wang)*.

There is so far no clear evidence for the existence of Martian life. But there are some potential signs of life. For example, seasonal variation of methane concentration has been observed on Mars. On the Earth, most methane is generated by organisms. This may also be the case on Mars. But, this seasonal change can also be explained entirely by abiotic chemical processes. So, whether there is or was life on Mars remains a grand open question to be answered.

***Wang:*** Prof. Pan's description is very comprehensive. Personally, I think the evidence is quite sufficient to conclude the existence of liquid water. Comparing remote sensing images of the same area taken at different times, we can see that some areas that were previously considered to be ice are more likely to be high-concentration brine water. It is also very likely that liquid water will be found underground. One of the main objectives of Tianwen-1 is to search for water-ice and liquid water.

## The mission of Tianwen-1

***NSR:*** Compared with previous Mars missions, what is special about Tianwen-1?

***Wang:*** China's lunar exploration project followed the three steps of orbiting, landing and returning. But Tianwen-1 is going to perform orbiting, landing and roving in a single mission, exploring Mars simultaneously from an overall global scale and a local fine scale. That is the highlight of Tianwen-1.

Specifically, the orbiter, or the satellite, will observe the whole planet of Mars, and the rover will perform detailed exploration of the landing and roving areas. This integrated space–ground observation strategy will provide us overall and detailed data of Mars, including its water-ice distribution, surface morphology, magnetic field, etc.

***NSR:*** What kinds of payloads are carried by Tianwen-1? What scientific results will be obtained?

***Wang:*** There are 13 scientific payloads, which can be categorized into four types: the optical cameras that will image the planet from both the orbiter and the rover; the radars that will detect the underground structures; the spectrometers that will analyze the composition of soils and rocks; and the monitors for atmosphere and space environments that will detect the magnetic field, space radiation and the climate of Mars.

Taking advantage of these payloads, we are going to obtain the following data and results. First, we will further map the Mars landscape and better understand how the planet was shaped by wind, water, volcanic activity and celestial body impacts. Second, we will learn about the distribution and reserves of water-ice and better understand the water escape process. Third, we will learn more about the composition, distribution and property of Martian soil, information helpful for our understanding of the evolutionary history of the Martian environment. Finally, we will get comprehensive and detailed data about the space and atmospheric environment of Mars.

***NSR:*** Among these possible results, which ones are you personally most interested in?

***Pan:*** Any data obtained by direct observation is of great value. There have been several successful Mars exploration missions, but generally speaking, direct observations of this planet are still very limited. The first-hand data from Tianwen-1 will enrich our understanding of Mars.

***Wang:*** That is right. I am personally interested in the magnetic field of Mars. Scientists speculate that there was once a global dipolar magnetic field on Mars, which is similar to the Earth's magnetic field that prevents the atmosphere from escaping and protects life from the solar wind. But at some point in history, the Martian magnetic field largely disappeared and Mars became an old, inactive planet. I hope to see more data about the evolution process of the Martian magnetic field.

***NSR:*** I noticed that there is no payload installed on the lander of Tianwen-1, which is different from the lander of China's lunar probe Chang’e-4. Why is that?

***Wang:*** The lander of Tianwen-1 is relatively simple. Its major function is to guarantee the safe landing of the rover. We did not design it for scientific observation and it is not capable of payload installation.

***NSR:*** Are you confident about the success of Tianwen-1? What are China's plans for further Mars exploration?

***Wang:*** I am confident. The confidence is not blind, but based on our solid preparations. There is no back-up probe for Tianwen-1, so in case of failure, we would not be able to repeat the same mission in 2022. But we have further plans to collect samples from Mars. Regardless of whether Tianwen-1 is completely successful or not, our Mars exploration program will continue.

## How is the mission organized?

***NSR:*** How were the scientific objectives and payloads of Tianwen-1 determined?

***Wang:*** China's lunar and deep-space exploration programs are planned in a systematic way. The China National Space Administration and the Chinese Academy of Sciences (CAS) jointly organized a national scientific committee to discuss and chart out the roadmap. The discussion was led by CAS, with the participation of more than 100 scientists from different research institutions and universities. We argued for many possible missions and finalized the roadmap. We put on hold the exploration of the Sun and Venus, and proposed detailed scientific objectives and implementation plans for the exploration of the Moon, Mars, the Jupiter system and the asteroids. In addition, we are also discussing the exploration of the heliospheric boundary of the solar system.

***NSR:*** Will this roadmap be further extended?

***Wang:*** Yes, of course. The current roadmap lasts up to around 2030. We will surely set up a new roadmap for the longer term. Moreover, besides this national roadmap, CAS also has its own funding programs for space exploration, which may be smaller but more innovative—for example, programs about Venus are under discussion.

***NSR:*** In November 2020, the Fourth Workshop for Scientific Payload Technology and Data Processing of Tianwen-1 was held in Chengdu. How does this series of workshops help the Tianwen-1 mission?

***Pan:*** These workshops bring scientists, engineers and administrators together to discuss all the related details. In the recent workshop, we focused on Tianwen-1’s payloads, discussed related scientific and technological issues regarding Martian geology, geophysics, space environment, etc. How to effectively use the data was also discussed.

**Figure:**
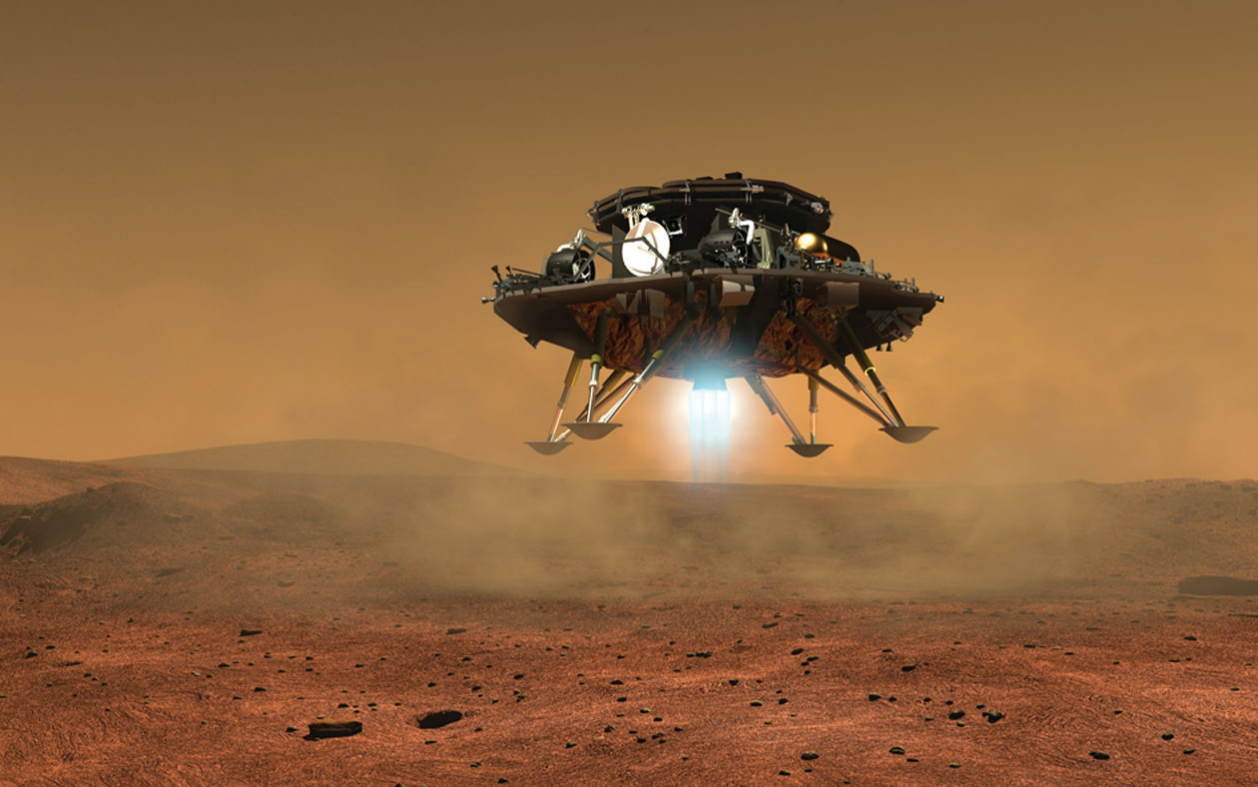
Artist's impression of the landing of Tianwen-1, the rover on the lander. *(Courtesy of the National Space Science Center, CAS)*.

***Wang:*** I attended the first three workshops of this series. I think these workshops can help in two aspects. Firstly, they provide a communication platform for administrators, engineers and scientists. The scientists need to understand the working mechanisms and performance parameters of the payloads made by engineers, otherwise they would not be able to correctly understand and analyze the data. Secondly, before we can really analyze the data from Tianwen-1, we need to do much preparative work, such as building the theoretical models and preparing the data analysis methods. These workshops can promote such scientific preparative work by bringing most experts together.

## Mars exploration plans around the world

***NSR:*** NASA’s Perseverance is also heading for Mars. Interestingly, Perseverance brought a helicopter with it. Will a helicopter become a common payload for the exploration of extraterrestrial planets?

**Wang**: The Mars Helicopter is a big innovation of Perseverance. The atmosphere of Mars is relatively thin, but still able to support helicopter flight. Helicopters can fill the gap of middle-scale observation between the large-scale observation of obiters and the local-scale observation of rovers. Compared to ground rovers, helicopters can go faster and farther. They can also access steep and complex landforms which cannot be reached by ground

rovers. So, I think the helicopter will be an important tool for future Mars exploration.

***NSR:*** NASA is very experienced in Mars exploration. What are their successful experiences that China can learn from?

***Wang:*** There are several important points. First, NASA has a long-term and stable roadmap for Mars exploration. They go step by step according to the roadmap. Stable funding and a long-term plan are the basic guarantees for a project as large as Mars exploration.

Second, their scientific objective is very significant and clear: to search for life on Mars. To answer related questions, they first followed the water, and then directly searched for possible signs of life. Moreover, the United States has a very strong group of planetary scientists, who have worked on Mars for decades and have a deep understanding of this planet. In China, we still lack real ‘Mars scientists’.

Third, NASA’s payload technology is very advanced. Compared to them, the performances of many of China's payloads need to be greatly improved.

***Pan:*** That is right. We do need a stable group of planetary scientists for Mars exploration. In past years, many Chinese researchers and educators, including the former Tianwen-1 Chief Scientist Weixing Wan (万卫星), have been promoting planetary science in China. We hope that planetary science can be recognized as a first-level discipline in college education, so that we can foster a group of planetary scientists for the next few decades of deep-space exploration and research activities.

I am reading Steve Squyres's book ‘Roving Mars’. He was the Principal Investigator of Spirit and Opportunity, and talked in his book about his experiences of communicating with engineers, about how they cooperated with each other to balance scientific inquiries and engineering concerns. We also need effective communication and mutual trust between scientists and engineers. I believe that we will do better in this aspect in the future.

***NSR:*** NASA also did a good job with data sharing. How about China?

***Pan:*** Data sharing was a main topic of the Chengdu workshop. We have to guarantee the reliability and standardization of data, and distribute the data to scientists in a timely manner. That is the expectation of both scientists and engineers.

***Wang:*** Currently, NASA is doing the best with regard to data standardization and publicity. I think there are two reasons behind this. First, the intellectual property protection policies are well-developed in the United States, so that the data can be properly cited by almost all end users. Second, their core research group is very strong and confident. They are confident that the major discoveries will be theirs, no matter the data are shared with others or not.

***Pan:*** Exactly. We hope that the data from Tianwen-1 will attract more scientists into our Mars program, and then these scientists can further promote the future Martian program, so that we will collect more data. This positive feedback will increase the overall level of China's Mars and planetary research.

***NSR:*** Three Mars probes were launched in 2020. Europe, Russia, India and SpaceX also have their own Mars exploration programs. What do you think about international competition and cooperation?

***Wang:*** With regard to scientific research, international cooperation is the general trend. Searching for extraterrestrial life and a ‘second home’ is the common dream of humanity. International cooperation can draw on the experiences of various countries and thus reduce the risk and the cost, and promote data sharing. So, I believe that although some competition does exist now, humans will join hands to explore the universe in the future.

***Pan:*** And China has always welcomed international cooperation.

## Personal experiences and expectations

***NSR:*** What are your personal feelings and expectations as a member of China's Mars exploration program?

***Wang:*** I participated in China's Mars program as early as 2007. At that time, Russia started the Phobos-Grunt mission, which planned to bring soil samples back from Phobos, one of the Martian moons. China joined this mission. We designed and built the Mars satellite Yinghuo-1, which would have headed for Mars orbit together with Phobos-Grunt. In 2011, the mission was launched by Russia's Zenit rocket, which soon lost control and never went further than the Earth’s orbit.

Now, China has developed our own large-scale launch vehicle Long March 5, as well as our own deep-space monitoring network, so that we can launch Tianwen-1 with every step within our own control. So, my strongest feeling over these years has been that we must rely on ourselves for the development of science and technology.

***Pan:*** On 23 July 2020, I, together with Prof. Wang and many colleagues, watched the launch of Tianwen-1 at Wenchang Spacecraft Launch Site. I was really excited watching the Long March 5 rocket rising to the sky and carrying Tianwen-1 to its long journey towards Mars. To me, being a member of the Mars exploration program is truly an honor and also a great responsibility. I would like to do my best, together with colleagues and collaborators, to accomplish the Martian research of Tianwen-1.

I do hope that Tianwen-1 will successfully complete all its missions of orbiting, landing and roving. I do hope that future missions to explore deep space will forge ahead smoothly, so that we can better understand Mars, as well as the universe, the Earth and ourselves.

